# Relationship between a High Mortality Rate and Extreme Heat During the Summer of 1999 in Hokkaido Prefecture, Japan

**DOI:** 10.2188/jea.12.254

**Published:** 2007-11-30

**Authors:** Dongmei Qiu, Takeo Tanihata, Hitoshi Aoyama, Toshiharu Fujita, Yutaka Inaba, Masumi Minowa

**Affiliations:** 1 Department of Epidemiology and Environmental Health, Juntendo University School of Medicine.; 2 Department of Epidemiology, National Institute of Public Health.

**Keywords:** mortality rate, hot weather, Hokkaido

## Abstract

In order to describe the relationship between mortality rate and extreme heat during the summer of 1999 in Hokkaido, we calculated the monthly age-adjusted death rates, average monthly mean temperature and average monthly high temperature for the years 1995 to 1999 in Hokkaido. The materials were derived from Statistics and Information Department, Minister’s Secretariat, Ministry of Health, Labour and Welfare of Japan, Statistical Bureau Management and Coordination Agency Government of Japan and the Japan Meteorological Agency. Trends in the monthly age-adjusted death rates and temperature changes in the same period were analyzed. The highest average monthly high temperature for August and September (28.8°C and 23.8°C, respectively) occurred in 1999; the similar trend was observed in the highest average monthly mean temperature. In August 1999, there were 14 days with highest temperatures of 30°C and over. The age-adjusted rate in August 1999 was significantly higher compared with those for the years 1995 to 1998 (p<0.01). We concluded that an unusually hot spell in 1999 was followed by a high mortality rate in Hokkaido.

## INTRODUCTION

More and more attention has been paid to the influence of high air temperatures on human health. Increases in mortality rates and their relation to high air temperatures have been disscused^1^^,^^2^^)^. According to the “Hokkaido News” newspaper, the number of days with temperatures of over 30°C in Hokkaido prefecture during the summer of 1999 was greater than those of previous years. As a result, the incidence of dehydration and heat stroke, especially in the elderly, was high^3^^,^^4^^,^^5^^)^. In this paper, we describe the sharp rise in mortality, particularly during the hot summer of 1999 in Hokkaido prefecture.

## MATERIALS AND METHODS

Population data for 1995 were derived from the 1995 Population Census of Japan; population data as of October 1 for the years 1996 to 1999 were estimated using information derived from the Vital Statistics of Japan. Mortality data were referred to the Vital Statistics of Japan (1995-1998) and the Monthly Report on Population Estimates (1999). Using these data, the monthly age-adjusted death rates were calculated using an indirect method. The average value of age-specific death rates for 1995 to 1998 in Hokkaido was employed as the standard. Changes in air temperature were analyzed using data from the Annual Report of Climatological Stations, published by the Meteorological Agency. The “average monthly mean temperature” refers to the average value of the mean temperatures for all days in the month; similarly, the “average monthly high temperature” refers to the average value of the highest temperature on all days in the month. The “average monthly mean temperature” and “average monthly high temperature” for Hokkaido were calculated by averaging the average monthly mean temperatures and the average monthly high temperatures for the cities of Sapporo, Obihiro, Hakodate and Asahikawa. Trends in the monthly age-adjusted death rates and temperature changes between 1995 and 1999 in Hokkaido, Japan, were then analyzed.

## RESULTS

### 1. Average monthly high temperature and average monthly mean temperature in Hokkaido, 1995-1999 (Table 1)

Between 1995 and 1999, the highest average monthly high temperature for July (26.6°C) occurred in 1997, and the highest average monthly high temperature for August and September (28.8°C and 23.8°C, respectively) occurred in 1999; similarly, the highest average monthly mean temperature for July (21.3°C) occurred in 1997, and the highest average monthly mean temperature for August and September (23.9°C and 18.8°C, respectively) occurred in 1999.

**Table 1.** Average monthly high temperature and average monthly mean temperature in Hokkaido, 1995-1999.

	Average monthly high temperature °C			Average monthly mean temperature °C		
	
	July	August	September	July	August	September
1995	24.9	24.1	21.3	20.7	20.5	16.5
1996	23.2	23.9	21.3	19.5	19.9	17.0
1997	26.6	23.3	20.6	21.3	19.3	16.2
1998	24.2	24.0	23.4	19.4	20.3	18.5
1999	25.8	28.8	23.8	21.1	23.9	18.8

### 2. Number of days between July and September with temperatures of 30°C and over in Hokkaido, 1995 -1999 (Table 2)

In July 1995 and August 1996, there were 3 and 1 day(s), respectively, where the temperatures at 30°C and over. In July and August, 1997, there were 8 and 2 days, respectively, with temperatures of 30°C and over. However, the highest temperature in July 1997 was only 31°C. In July 1999, there were 6 days with temperatures of 30°C and over. In August 1999, there were 14 days with highest temperatures of 30°C and over, the largest number for any month examined in this study. During this month, the temperature reached 31°C on two days, 32°C on one day, 33°C on three days, and 34°C on four days. No temperatures of 30°C and over were recorded for August and September 1995, July and September 1996, September 1997, July to September 1998, or September 1999.

**Table 2.** Number of days with highest temperatures between July and September in Hokkaido, 1995-1999.

	°C			

	<19	20-24	25-29	≥30
July				
1995	1	12	15	3
1996	2	18	11	0
1997	0	7	16	8
1998	2	15	14	0
1999	1	11	13	6
August				
1995	0	14	17	0
1996	0	16	14	1
1997	6	13	10	2
1998	1	18	12	0
1999	0	4	13	14
September				
1995	7	21	2	0
1996	3	26	1	0
1997	13	13	4	0
1998	2	16	11	0
1999	4	12	14	0

### 3. Secular trends in age-adjusted death rates (Fig. 1)

Of the age-adjusted death rates for the months of July and August between 1995 and 1999 in Hokkaido, the highest rates occurred in 1999 (7.1‰ in July, 7.5‰ in August). The age-adjusted death rate for August 1999 was particularly higher than any rate in August between 1995 and 1998. Difference between August 1999’s age-adjusted rate and other rates measured during above mentioned period appeared to be all statistically significant at almost same level (p<0.01). For the month of September, 1995 to 1999, the highest age-adjusted death rate occurred in 1995 and 1999 (7.1‰).

**Figure 1.**
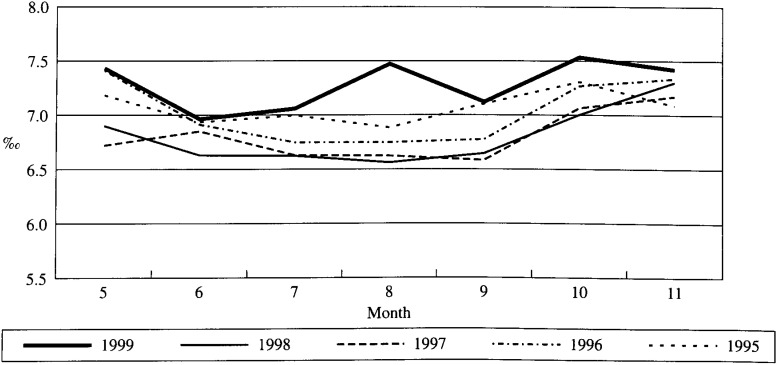
Age-adjusted death rates between July and September in Hollaido, 1995-1999.

## DISCUSSION

The occurrence of heat stroke was significantly correlated with a high temperature of over 30°C^6^^)^. Death rates from no particular cause were related not only to an increase in temperature, but to the number of continuous days with high temperatures^2^^)^. The relationship between the number of deaths from heat stroke and the number of days with temperatures of over 30°C was significant, according to accumulated over a 21-years period between 1970 and 1990 in Japan^2^^)^.

In this study, the highest average monthly high temperatures occurred in August and September of 1999. In August, the temperature was 30°C and reached over 30°C on nearly half the days of the month (14 days). The mortality rate gradually increased in July, August and September of 1999, peaking in August.

The human body dissipates heat in four ways: convection, conduction, radiation and water evaporation. When the temperature of the surrounding environment is higher than the body temperature, water evaporation is the only way in which the body can dissipate heat. When the air temperature is over 34°C, cardiovascular and nervous disorders can occur as a result of problems associated with body temperature adjustment and the metabolism of water and salts^7^^,^^8^^)^.

Hokkaido is located in the most northern prefecture of Japan. Its climate is usually pleasantly cool during the summer. Between 1961 and 1998, the average monthly temperatures in Sapporo (the capital of Hokkaido prefecture) were 22.0°C, 24.0°C, and 20.2°C for July, August and September, respectively^9^^)^. Unexpectedly high temperatures are a source of substantial heat stress to residents, who are accustomed to a cool climate. Unexpectedly high temperatures are particularly difficult for the elderly^10^^,^^11^^)^, and people with chronic diseases^12^^,^^13^^)^. A high mortality rate during a 1980 heat wave was also reported by Jones^10^^)^. One important reason for the high mortality rate in the elderly during periods of hot weather is thought to be a reduction in heat adaptation caused by use of some drugs commonly prescribed to the elderly. High temperatures were found to lead to a deterioration in the condition of elderly people with common diseases, leading to their death^14^^)^. The mortality rates from heat stroke in the elderly also rose in the summer of 1988 in China^13^^)^.

In hot weather, having a working air-conditioner or visiting an air-conditioner place can reduce heat-related death^12^^)^. The number of room air-conditioner (109/1,000 households) in Hokkaido was much less than that in Kanto area (2,155/1,000 households) in 1999^15^^)^. It is suggested that the equipment against the heat was inadequacy when the average air temperature in Hokkaido was similar to that in the metropolitan area in the summer of 1999^3^^)^.

In the present study, we observed that, August 1999, was a particularly hot month in Hokkaido, with the number of days with temperatures of 30°C and over and the average monthly temperature being much higher than usual. The age-adjusted death rate was also much higher than usual. Although the mortality rates for specific ages groups, gender, and causes were not examined in this study, we expect that the number of deaths from heat stroke can be decreased by increasing water intake and improving housing conditions, etc., especially in groups who are at risk for heat stroke. The data used in this study was obtained from the Monthly Report of Vital Statistics, so the influence of other factors, such as age, gender, cause of death, and region could not be analyzed. Further research will be performed when information on these factors becomes available.
